# Collaborative Implementation Planning to Jumpstart Advance Care Planning with Alaska Native and American Indian peoples in Primary Care

**DOI:** 10.21203/rs.3.rs-7400899/v1

**Published:** 2025-09-19

**Authors:** Rajinder Sonia Singh, Sara J. Landes, Shannon Medlock, Christopher Piromalli, Ryan Mortenson, Jennifer Shaw

**Affiliations:** University of Arkansas for Medical Sciences; University of Arkansas for Medical Sciences; Southcentral Foundation; Southcentral Foundation; Southcentral Foundation; University of Alaska Fairbanks

**Keywords:** Alaska Native/American Indian Peoples, Advance Care Planning, Implementation Science, Health Equity

## Abstract

**Background:**

Palliative care, including advance care planning (ACP), ensures a patient’s medical care aligns with their values, goals, and priorities throughout serious illness or injury. The Alaska Native and American Indian (AN/AI) population is greatly increasing and less likely to have documentation on ACP conversations. AN/AI peoples are more likely to engage in ACP when it is culturally-tailored to their specific needs. Jumpstart AN/AI is a culturally-tailored tool to assist providers and customer-owners with starting ACP conversations. We conducted a qualitative study to gather feedback from customer-owners and employees to inform implementation of Jumpstart AN/AI within primary care at Southcentral Foundation (SCF), a Tribal health system.

**Methods:**

We conducted a qualitative, descriptive study using a community-based participatory research approach. Semi-structured interviews and focus groups were conducted with customer-owners (n=14) and SCF employees (n=16). We used template analysis, a rapid qualitative data reduction technique, to analyze results from the qualitative interviews and focus groups. Results were then presented to the research team and SCF leadership to finalize implementation plans for Jumpstart AN/AI within SCF.

**Results:**

All participant groups were in favor of implementing Jumpstart AN/AI and expressed the importance of ACP conversations for AN/AI peoples. SCF employees stated Jumpstart AN/AI delivery would need to be flexible and meet the needs of customer-owners as well as providers and staff. Customer-owners reported they trust their care teams to deliver Jumpstart AN/AI and would also value patient-facing materials so they could ask their provider about Jumpstart AN/AI.

**Conclusions:**

Qualitative data collected from SCF employees and customer-owners, rapid template analysis, and collaboration with Tribal health system leaders resulted in successfully and quickly developing a flexible implementation plan to integrate Jumpstart AN/AI into the Tribal health system. Implementation of Jumpstart AN/AI will contribute to the limited evidence base for AN/AI-tailored ACP interventions and inform implementation research and practice.

For clinical trials, the trial registry name and URL, and registration number must be included at the end of the abstract.

## Background

Palliative care is an interdisciplinary medical specialty focused on maximizing quality of life throughout serious illness or injury (1–3). Advance care planning (ACP), a key component of palliative care, specifically focuses on aligning care with individual patient’s values, goals, and priorities. ACP conversations help patients with serious illness understand potential care trajectories and prepare for future treatment decisions. It is ideally documented in the patient’s electronic health record (EHR) as advance directives (AD)(3) that specify patient preferences for life-sustaining care (e.g., intubation) and surrogate decision-makers to guide care when patients cannot speak for themselves. For patients and their families, ACP can decrease depression (4), anxiety (5), grief (6); and unwanted, non-beneficial treatments (7, 8). For healthcare providers and systems, ACP results in better support for patient care, reduced costs, and improved care quality (9). While having serious illness increases the likelihood of having an AD, 67% of people with chronic illness do not have one (10). ACP is often introduced, if at all, too late in an illness for patients, their families, and health care providers to benefit (11, 12).

The need for high-quality ACP in the Alaska Native and American Indian (AN/AI) population is growing. The elder AN/AI population is expected to triple between 2010 and 2050, with a disproportionate burden of serious illness (13–18). Despite a clear need for ACP, AN/AI peoples are less likely than other demographic groups to have ACP conversations documented in the EHR (19, 20). In one study, AN/AI peoples were half as likely as their White peers to have ADs, an indication of an ACP conversation, when controlling for individual factors, such as spiritual and religious practices (21). In addition, large database studies have showed that AN/AI peoples are less likely to have ADs ordering Do-Not-Resuscitate (DNR) or Do-Not-Hospitalize (DNH) (22, 23). Limited use of palliative care among AN/AI peoples has previously been attributed to cultural barriers, such as prohibitions on talking about death (24). More recent research has found that AN/AI peoples are as likely as other groups to use ACP when it is available and offered in a culturally appropriate way (21, 25). To date, however, few culturally tailored ACP interventions have been available to AN/AI patients and their health care providers.

To address the lack ACP in AN/AI communities, our team conducted a study to culturally tailor and pilot an evidence-based communication intervention to promote ACP communication between AN/AI patients with serious illness, their family members, and providers (26). The selected intervention, Jumpstart, occurs in 3 steps: (1) the patient completes a brief questionnaire, that can be self-administered, to identify individual preferences for life-sustaining care and perceived barriers and facilitators for ACP communication; (2) this information is used to create an individualized, one-page ‘tip sheet’ (one for the provider and one for the patient) with personalized tips for starting an ACP conversation; and (3) the provider and patient are given the tips sheets to prompt an ACP conversation at the next clinical visit. While Jumpstart was found to significantly increase ACP conversations and documentation, compared to usual care, as well as patient-rated quality of communication (27), in two clinical trials, until recently it had not been implemented in routine care or tailored for use with AN/AI patients.

In our first Jumpstart study, we used a community-based participatory research approach (28) to culturally tailor and pilot Jumpstart for use with diverse AN/AI peoples with serious illness (28, 29). We partnered with two Tribal health systems to culturally tailor the intervention and then piloted the adapted intervention, Jumpstart AN/AI, with 58 AN/AI adults living with serious illness. The pilot showed high acceptability of the intervention and feasibility of a larger trial, as well as signs of effectiveness. Nearly all (95%) participants reported Jumpstart AN/AI helped them to have ACP conversations with their primary care providers, and new ADs in the EHR were 3.5 times higher in the treatment group than the control group, suggesting Jumpstart AN/AI increased ACP conversations and documentation (29). However, the pilot was not designed to evaluate comparative effectiveness.

Our current study aims to evaluate Jumpstart AN/AI, as compared to usual care, in a type 1 hybrid effectiveness-implementation (30–32) cluster randomized controlled trial (cRCT) to establish evidence for effectiveness and gather data on implementation of the tailored intervention. Prior to launching the cRCT, we conducted a qualitative study to gather feedback from AN/AI adults with serious illness, providers, and leaders to inform implementation of Jumpstart AN/AI within in the Tribal health system. This paper presents results of that study.

## Methods

We conducted a qualitative, descriptive study using a community-based participatory research approach in which AN/AI individuals were directly and substantively involved in co-creating, conducting, and approving the research design, protocol, and dissemination. Semi-structured interviews and focus groups were conducted to elicit stakeholder perspectives about implementing Jumpstart AN/AI at Southcentral Foundation (SCF), a large Tribal health organization in Alaska. Findings from the focus groups and interviews were used to inform decisions about implementation and randomized controlled trial (RCT) design. All research activities were approved by Southcentral Foundation Board of Directors, the Alaska Area Institutional Review Board, and the University of Arkansas for Medical Sciences Institutional Review Board. All research activities were conducted following ethical guidelines including those outlined in the Declaration of Helsinki as revised in 2013.

### Research Team Positionality

More than half of our research team was comprised of AN/AI individuals working within SCF as research, clinical, or process improvement staff. Of the non-AN/AI researchers, two had several decades of combined experience conducting research or providing clinical care within AN/AI communities. The remaining non-AN/AI researchers had experience working with rural populations, were new to working with AN/AI communities, and had extensive implementation science expertise. We met virtually no less than monthly and in person annually for two days to reflect on and plan study activities, and to foster shared awareness of the study setting and cultural context for enhanced rigor and validity of findings. These visits included activities for team members to learn about AN/AI cultures and the Alaska Tribal Health System.

### Setting

This study was conducted at Southcentral Foundation (SCF), an Alaska Native-owned, nonprofit health organization serving nearly 70,000 AN/AI peoples in Anchorage, the Matanuska-Susitna Valley, and 55 rural villages. SCF is one of 30 Tribal health organizations that comprise the Alaska Tribal Health System, serving about 180,000 people total. People receiving care at SCF are called customer-owners because, as enrolled members of the Tribes that own and operate the health system, they are both ‘customers’ and ‘owners’ of that system. SCF operates two large urban primary care centers, with 11 primary care clinics combined, as well as 14 rural clinics spread over 107,400 miles. A total of ~ 45 primary care providers (MD, DO, PA, NP) lead multidisciplinary integrated care teams, with co-located specialists in palliative care, pain management, pharmacy, and behavioral health. SCF operates on a “patient-centered”, medical home model called Nuka System of Care that prioritizes relationships, family wellness, and shared responsibility between customer-owners and care providers.

### Participants and Procedures

#### SCF Employees.

We conducted interviews with SCF employees to elicit feedback on Jumpstart AN/AI implementation. Employee interviews were conducted prior to customer-owner interviews to identify potential strategies for implementation and ensure that customer-owners were only asked for feedback about options that were feasible within the health care system. We used purposive sampling to recruit a variety of SCF employees with experience relevant to ACP delivery, including clinicians, information technology specialists, and clinical and operational leaders within primary care and other departments. Employees were eligible to participate if they were employed by SCF for at least one year, directly or indirectly supported primary palliative care delivery, and had expertise and/or experience pertinent to development or delivery of primary care services. We recruited employees through direct emails, and all participating employees provided informed consent. Employees could not be compensated for interviews conducted during working hours per organizational policy.

#### Customer-Owners.

We conducted focus groups and qualitative interviews with customer-owners to gather feedback about potential options to deliver Jumpstart AN/AI identified through the employee interviews. We recruited customer-owners through SCF social media posts and flyers posted in clinics, as well as through tabling events in the primary care center lobby. Customer-owners were eligible to participate if they were at least age 50, had at least two primary care visits in the previous 12 months, and had one or more serious illness (e.g., cancer, lower respiratory disease, diabetes). All customer-owners who took part in the study provided informed consent and received a $50 gift card.

### Data Collection

The updated Consolidated Framework for Implementation Research (CFIR) was used to guide data collection (33). CFIR is an implementation determinants framework designed to identify factors believed or empirically shown to influence implementation of interventions or programs. The five domains of CFIR include 1) innovation domain (i.e., the “thing” being implemented), 2) outer setting domain (e.g., hospital system, school district, state), 3) inner setting domain (e.g., primary care clinic, classroom), 4) individuals domain (i.e., the roles and characteristics of individuals involved), and 5) implementation process domain (i.e., the activities and strategies used to implement the innovation). We used CFIR domains to develop semi-structured guides for both employee and customer-owner interviews.

#### SCF Employees.

Employee interviews were conducted from December 2023 to February 2024. Interviews lasted approximately one hour each and were conducted by video conference. Employees were asked about their role at SCF, experiences with and opinions about ACP, their initial reactions and opinions about the Jumpstart AN/AI intervention, thoughts on how customer-owners should complete the patient questionnaire, how and when customer-owners should receive their feedback form, how and when providers should receive their feedback form, main considerations to address before implementation, how to measure successful implementation of the intervention, and best indicators of clinical effectiveness of Jumpstart AN/AI. See Appendix 1 for the employee interview guide. After 11 interviews, the study team removed the questions about how to measure successful implementation of an intervention and best indicators of clinical effectiveness of Jumpstart AN/AI as outcomes were already identified in the project proposal and participants often suggested the study team would be the best to answer those questions.

#### Customer-Owners.

We initially conducted focus groups with customer-owners (n = 9), followed by several qualitative interviews (n = 5) to address questions raised in full-team discussions about the preliminary results. Focus groups were conducted in April 2024 and qualitative interviews occurred in July 2024. Focus groups lasted approximately 1.5 hours and qualitative interviews lasted approximately 30 minutes; both were conducted in person. Customer-owners were asked why they chose to participate in the study, their opinions about ACP, their thoughts on how customer-owners should complete the questionnaire, their thoughts on how customer-owners should receive the feedback form, and any additional perceptions about ACP or the Jumpstart AN/AI intervention. See Appendix 2 for the customer-owner interview guide.

### Data Analysis

All interviews and focus groups were recorded, transcribed verbatim, and rapidly analyzed using template analysis, a data reduction technique developed by health services researchers (34). Template analysis is useful for using data to inform health service implementation in a short timeframe. The lead analyst reviewed the interviews and developed domains, subdomains, and categories based on interview and focus group content. Categories were developed based on participant responses (e.g., competing demands within the health care system). Although domains, subdomains, and categories were developed a priori based on the interview guides, they were modified as the analysis was conducted. Analysis teams established consistency, templated the remaining interviews, and collaboratively reconciled discrepancies. After all interviews and focus groups were completed, the lead analyst created a template matrix that included domains, subdomains, and categories which could be viewed by participants to examine individual differences in responses.

### Implementation Planning

Following preliminary data analysis, the entire study team met for two days in May 2024 to review the qualitative results and begin tailoring a plan to implement Jumpstart AN/AI for the clinical trial. The study team was intentionally assembled to include individuals with expertise in palliative care/ACP, Tribal health services, community-engaged research, clinical trials, statistics, qualitative research, process improvement, the Jumpstart intervention, and implementation science. The lead analyst presented the data findings and outlined for the group several options for key implementation decisions.

The group then worked through a process to make key decisions for implementing Jumpstart AN/AI in the context of an RCT; see Table 1. Discussion was guided by the Behavioral Health Quality Enhancement Research Initiative (QUERI) implementation planning guide template (35).

## Results

A total of 30 people participated in the study, including 14 customer-owners and 16 employees. Table 2 provides an overview of participant characteristics. Most (56%) employee participants worked in primary care (56%), were over 40 years old, identified as White (63%) and female (62%), and worked as operational and clinical leaders, clinicians, and support staff, community resource specialists, physical therapist, and other professionals. Customer-owners ranged in age from 51–78 (M = 63, SD = 7.9). All customer-owners identified as AN/AI. Customer-owners were mostly female (86%) and had college/or higher education (43%). Results are presented below by theme. Quotes that represent the theme are provided, but as with qualitative methods these are not intended to reflect the words and thoughts of all participants.

### Perceptions of Implementing ACP and Jumpstart AN/AI

#### SCF Employees

Most employee participants supported implementing Jumpstart AN/AI in the health system. SCF employees liked that it was concise, it shared the same information to providers and customer-owners, and it was personalized to the individual customer-owner.

“I think the idea behind it which is a customized conversation around the goals and wishes of the customers - iťs what we should be doing. We should be engaging at a level of what matters for the customer at every visit. And this is another way to be able to do that around [ACP], which is great.” (Employee 06)

Employees discussed the importance of context in timing and delivery of Jumpstart AN/AI and ACP conversations generally. One employee described, “I think it would just depend on how the surveys are given out because it could be a little bit startling to people who are not ready for that conversation.” (Employee 15).

#### Customer-Owners

Several customer-owners discussed that ACP conversations are important to ensure their wishes are known and that their families have a plan for their end-of-life care. One customer-owner said, "I think iťs important to plan ahead for those situations, so my children donť have to make difficult decisions for me." (Customer-Owner 01).

Like employees, customer-owners discussed the importance of timing of ACP conversations and emphasized the need for provider sensitivity to the customer-owner’s individual circumstance and their willingness to engage in the conversation.

“Someone did approach me about doing an advanced directive about three months ago, and I kind of felt offended. Iťs like you’re kind of – you want me to plan for in advance for my death, and I’m not worried about that right now. I’m worried about other things, you know. So, it – time appropriateness.” (Focus Group 01)

#### Barriers and facilitators to Implementing Jumpstart AN/AI and ACP

Participants identified a wide range of barriers and facilitators to implementing Jumpstart AN/AI in the Tribal health system and ACP in general; see Table 3. Below we expand on most relevant barriers and facilitators.

#### Barriers to Implementing Jumpstart AN/AI and ACP

Most of the barriers were related to the health care system with employees noting barriers related to competing demands, staffing shortages, and how to integrate information into the health care system.

“We have many, many screeners, so the challenges around it are that here’s one more thing that our teams need to know - who is supposed to be administered which screener. And then, once you get the information, whaťs going to be done with the information once the screener is done, and how do you fold this into a system thaťs already burdened.” (Employee 06)

Similarly, one employee noted the overall staffing shortages, “We also have a shortage of MAs and a shortage of behavioral health consultants right now. We are pretty thin on staffing […] just kind of staying afloat the best that we can, keeping our heads above water.” (Employee 07)

Employees indicated that providers may be uncomfortable bringing up ACP with customer-owners. SCF employees stated this could be potentially due to their concerns of how it will be received by customer-owners, and one described ACP as a “touchy subject.”

#### Facilitators to Implementing Jumpstart AN/AI and ACP

SCF employees reported they perceive that customer-owners trust the health care system and their providers. For example, one participant noted how they wait until they build trust with patients before engaging in ACP conversations, “I’ve found after getting to know somebody over time and building up trust, especially when someone’s health status is changing for the worst [I] bring up concerns and ask them what they care about. ” (Employee 20).

Similarly, customer-owners expressed having trust in the health system and their primary care team. One customer-owner described a decades long relationship with their primary care provider, “I speak with my doctor. She’s great. She’s been my doctor for I want to say 25, 30 years.” (Focus Group 02).

SCF employees described being comfortable with conversations related to ACP and their own trust building practices that may facilitate ACP in general and implementing Jumpstart AN/AI.

“I continue to engage patients in [ACP] conversations, especially after I’ve gotten to know someone and I’ve built a relationship, because these conversations can be really emotional, for people and [bring up a lot]. I’ve found after getting to know somebody over time and building up trust, especially when someone’s health status is changing for the worst to share my concerns and ask them what they care about, what matters to them, and if they couldnť speak for themselves who would be a voice for them for their concerns.” (Employee 12)

This focus on developing trust and provider comfort matched patient’s comfort with ACP conversations. For example, one stated, “I have no problem talking about dying and death…” (Customer-owner 05). Further, some customer-owners stated they were comfortable using the patient portal and were familiar with completing regular screenings and questionnaires within the health care system. Customer-owners discussed the importance of support from their family and their AN/AI communities.

#### Supports needed to Implement Jumpstart AN/AI

Employees emphasized the need for multiple, flexible methods of delivery of Jumpstart AN/AI to maximize intervention reach, noting that paper, technology-facilitated, and/or face-to-face delivery may be needed for a customer-owner population that is diverse in cultural backgrounds, age, illness type and severity, and other life circumstances. SCF employees highlighted delivering Jumpstart AN/AI using methods that are most acceptable and appropriate to the different needs of customer-owners. Customer-owners emphasized that implementation should incorporate culturally centered strategies such as engaging family, learning circles, and shared story.

#### SCF Employees

SCF employees suggested offering customer-owners multiple delivery options and providing concordant support, “I’m just imagining if a customer’s filling [the questionnaire] out in the exam room that there’s somebody checking back in on them. You know, cause some of these questions I think could also be triggering for the customer. And if iťs filled out at home, maybe a follow up call to see how the person’s doing and what questions they may have on the form and/or how did it, how was their experience filling it out?” (Employee 04).

We asked SCF employees about the best methods to deliver the Jumpstart AN/AI intervention including: 1) how should the customer-owner complete the questionnaire, 2) how should the customer-owner-facing tip sheet be delivered, and 3) how should the provider-facing tip sheet be delivered. Based on input from SCF employees and the need for various, flexible methods, we created two options that were discussed with customer-owners, leadership, and presented to the research team (Table 4).

#### Customer-Owners

Customer-owners discussed the need for Jumpstart AN/AI to match their specific cultural needs including incorporating family members, completing the intervention in settings that are most acceptable to them (e.g., learning circles or one-on-one, depending on preference), and incorporating customer-owner story.

“But iťs like our whole family goes through my mother’s doctor’s appointments. We’re all piled in. Iťs all involved. We’re all involved. We all have a say. We all have our input and iťs strong within us. [...] And if we have like maybe a navigator when the family’s there to make the decisions together with the person who’s ill is – oh, I think thaťd be great.” (Focus Group 01)

Customer-owners also discussed how helpful certain other information campaigns were within SCF (e.g., diabetes screening) and suggested following those models of communication and engagement. They highlighted how helpful having customer-owner facing materials were for them so they could be actively engaged in their own health care and empowered to ask questions of their health care team. Of note, several participants (both employees and customer-owners) recommended using learning circles to implement the Jumpstart AN/AI. Learning circles are a culturally-centered group format, where people come together to discuss a topic of interest and/or complete a shared activity (e.g., cooking a meal together). SCF offers a variety of learning circles, ranging in focus from family wellness to diabetes management to other community-prioritized health topics.

#### Key Decisions made During Implementation Meeting

Based on the qualitative results, feedback from leadership, and implementation workgroup discussions, the study team, including SCF improvement and clinical staff, made key decisions about implementing Jumpstart AN/AI in the context of an RCT. First, given the enthusiasm in the health system for Jumpstart AN/AI, we discussed potentially moving from a hybrid type 1 design (with a primary focus on effectiveness and secondary focus on implementation) to a hybrid type 2 design (with an equal focus on effectiveness and implementation). The team discussed using the barriers and facilitator data to identify implementation strategies that could be piloted in a hybrid type 2 trial.

Some potential implementation strategies (36) identified included conducting ongoing training to address staff turnover/new staff, identifying a clinical champion, leveraging the EHR and patient portal for intervention delivery, administration, and recording, and marketing ACP information to customer-owners to raise awareness. Given the challenge of integrating in the EHR and preliminary nature of the implementation project, the team determined that the strategies that are most likely needed would be potentially challenging to use at this time. The findings related to change fatigue and staff turnover ultimately led to the decision to retain the hybrid type 1 design. The team came to the consensus that using an implementation strategy would be more resource intensive than what the health care system had agreed to do for the study or was available at this time. In addition, the team concluded that the primary goal of the study remained the need to establish evidence of Jumpstart AN/AI effectiveness. Therefore, we made the decision to conduct an RCT with a process evaluation (type 1) and not a type 2 (RCT + testing a bundle of implementation strategies). See Table 5 for decisions made.

## Discussion

The goal of this qualitative study was to elicit the perspectives of customer-owners and employees in an Alaska Native health system to inform the implementation of Jumpstart AN/AI in a cluster randomized clinical trial to evaluate intervention effectiveness and identify key factors for implementation. In addition to semi-structured, qualitative individual and group interviews conducted with customer-owners and employees, we engaged health system leadership, identified additional operational and clinical leader-champions in the health system to inform trial design and intervention delivery, and formed an implementation workgroup to finalize an implementation plan for the RCT.

This study found substantial support among both SCF customer-owners and employees for implementing Jumpstart AN/AI into primary care services. SCF employees had generally positive views of ACP and Jumpstart AN/AI, acknowledging that primary care staff are largely familiar with ACP and comfortable having ACP conversations. A few employees discussed discomfort in ACP conversations or concerns about how these conversations may be perceived by customer-owners. SCF employees identified supports needed to implement Jumpstart AN/AI that included engaging key members of the health care system and providing flexible methods of delivery for customer-owners, to accommodate a wide range of preferences for engaging with health services (e.g., individualized versus group visits, paper versus electronic formats) (37).

SCF customer-owners were also broadly in favor of implementing Jumpstart AN/AI and strongly supported an increase in ACP being offered in the Tribal health system. They reported a desire for the health system generally and primary care providers in particular to initiate ACP conversations more consistently and frequently, particularly with individuals and families that are experiencing serious illness. Like the employee participants, most customer-owner participants indicated that facilitators for implementing Jumpstart AN/AI included generally high trust in the Tribal health system and their provider teams, as well as a willingness to discuss ACP. This suggests that hesitance to discuss ACP may be more common among providers and staff than customer-owners, due to workload and/or relationship concerns (21, 25).

Barriers to implementing Jumpstart AN/AI were also identified among customer-owners, including a perceived community-wide lack of awareness about ACP and how it can support individuals and families during serious illness. Other barriers include limited engagement with the patient portal and technology in general among some customer-owners, and limited Alaska Native language interpreters in the health system (25). Of note, these data were collected while the health care system was still experiencing the impact of the COVID-19 pandemic. Since the time of data collection, SCF has worked to increase staffing levels in order to reduce burden on the system and improve employee well-being. SCF is also a learning health system in that it is consistently practicing quality improvement methods and attempting to implement changes to better serve Customer-owners and SCF employees (many of whom are also Customer-owners).

In considering supports needed to implement Jumpstart AN/AI, customer-owners offered suggestions for tailoring implementation strategies to individual preferences (e.g., group and one-on-one delivery) and cultural context (e.g., engaging family and community) (37). Customer-owners discussed the importance of community awareness campaigns and how these inform and empower customer-owners to advocate for their own care, which aligns with SCF’s core principle of ‘shared responsibility’ between customer-owners and the health system. This finding also highlights that engaging end-users in implementation efforts can simultaneously support individual and institutional needs and goals and facilitate increased health equity and efficiency in care delivery (38).

Some of the perceived barriers and facilitators to implementing Jumpstart AN/AI and ACP may appear to be contradictory. For example, some providers reported perceiving that customer-owners trust the health care system while others brought up perceived potential mistrust. We believe this highlights that providers are sensitive to how important trust is to the clinical encounter. Further, customer-owners reported they trust the health care system and their providers. The providers who brought up potential concerns for mistrust recognize the importance of trust and understand the culture of the health care system in achieving health through relationship. This sensitivity to the potential of mistrust highlights how providers attempt to approach the clinical interaction in a way that is thoughtful of historical marginalization and culturally tailored. We believe this thoughtfulness is then reflected in the customer-owner experience of reporting trust in the health care system.

Another example of potentially contradictory findings are SCF employees both reported a personal comfort with ACP conversations and the potential for employee discomfort with ACP conversations. Most of the SCF employees interviewed for this project highlighted they were comfortable with conversations around ACP. However, most stated this was due to training or experience. They noted their perceptions that other providers who may be newer or not have completed training may not be as comfortable or experienced with ACP conversations. These findings highlight the nature of qualitative research in which participants may highlight several different perceptions and ideas which taken together can paint a picture of perceptions within a setting.

The study team considered learning circles as a strategy for implementing Jumpstart AN/AI, but ultimately decided against this. In keeping with the transtheoretical model of health behavior change (39), the goal of Jumpstart AN/AI is to *prime an individual for an ACP conversation* (contemplation), and the learning circle strategy would be more appropriate for *enacting the ACP conversation* (action). However, the team agreed that future study of whether/how to integrate Jumpstart AN/AI into an ACP learning circle is warranted, as some health systems have begun doing group ACP visits (40).

### Implications

As described above, the study team used both the qualitative results, feedback from health system leaders, and an implementation workgroup consisting of researchers, clinicians, and healthcare improvement staff to make decisions for implementing Jumpstart AN/AI in the context of an RCT. These decisions, described in Table 5, will facilitate initiation of an RCT to evaluate the effectiveness of Jumpstart AN/AI for increasing ACP conversations in the Tribal health system, while facilitating collection of additional data on barriers and facilitators for implementation to inform future research and practice. One of the challenges the study team encountered was considering whether to continue with the plan for a type 1 hybrid effectiveness-implementation approach or change to a type 2 hybrid design. This potential change and resulting discussion highlight the challenge of determining what type of hybrid is the best fit for the data and context in which a study is being conducted. We feel that this challenge often occurs but is infrequently documented.

We intentionally formed a research team that included people with expertise as customer-owners, Tribal health researchers, health care providers, healthcare improvement specialists, implementation scientists, and clinical trialists to ensure that we developed the Jumpstart AN/AI implementation plan with consideration of and sensitivity to the needs, resources, and constraints of both the health system and the research study. A forthcoming protocol paper will describe in detail the final plan for implementing Jumpstart AN/AI in the context of a type 1 hybrid effectiveness implementation cluster randomized trial in the Tribal health system.

### Strengths and Limitations

The considerable strengths of this study include a community-based participatory approach in which customer-owners of the health system were at the center of the implementation questions. Longstanding engagement with the health system, as embedded and external researchers facilitated formation of a multidisciplinary research team that included customer-owners, clinicians, and improvement experts, which helped to ensure the validity of the data and the resulting interpretations. We began with SCF employees to ensure suggestions were implementable within the Tribal health system. Using template analysis allowed the team to conduct a rigorous, rapid analysis of the qualitative data so that the results could quickly inform implementation planning, including the collection of additional data and conversations with health system leadership. Finally, a strength of this project is that it provides an example of an implementation study team deciding between a type 1 or type 2 hybrid effectiveness-implementation approach. Implementation researchers may sometimes be uncertain of which type may best fit their needs and it is our hope that this paper provides a blueprint for considerations when making key decisions in study types.

Limitations to the study include the limited sample size and potential for limited perspectives. SCF employees and customer-owners in this study generally expressed a positive perception of ACP. While we have interpreted this to reflect widespread support for ACP services in the health care system, it is possible that there are countering views that were not captured during interviews due to self-selection bias. Customer-owners and staff who are not comfortable with talking about ACP may not have elected to participate in the study. A majority of the customer-owners who participated were women, highlighting that information from other genders may be limited.

## Conclusions

Qualitative data collected from SCF employees and customer-owners and collaboration between Tribal health system leaders, clinicians, and improvement experts resulted in successfully developing an implementation plan to integrate Jumpstart AN/AI into primary care. Implementation of Jumpstart AN/AI in the Tribal health system in the next phase of this study will contribute to an emerging evidence base for AN/AI-tailored ACP interventions and generate data to inform future Jumpstart AN/AI implementation research and practice. Collectively, these activities will help meet the rapidly rising need for high-quality, culturally appropriate ACP services to help ensure that AN/AI peoples and their loved ones want and need early and throughout the experience of serious illness.

## Supplementary Material

Supplementary Files

This is a list of supplementary files associated with this preprint. Click to download.

• Appendix1EmployeeInterviewGuide.docx

• Appendix2CustomerOwnerInterviewGuide.docx

## Figures and Tables

**Table 1:** Key decisions for implementing Jumpstart AN/AI in the context of an RCT.

• Process for customer-owner to complete ACP questionnaire • Process for populating customer-owner ACP conversation tipsheet (e.g., Access database, EHR build) • Process for populating provider tip ACP conversation tipsheet (e.g., Access database, EHR build) • Process and method for sharing customer-owner ACP conversation tipsheet • Process and method for sharing provider conversation tipsheet • Communication plan with operational partners and clinical teams prior to rollout

**Table 2:** Participant characteristics.

Employees (n = 16)		% (n)
Length of SCF employment	Range: 3–32 yrs; M = 13.69, (SD = 8.19)	
Position	Operational/Clinical Leader	19
	Primary Care Provider	19
	Behavioral Health Professional	13
	Certified Medical Assistant	13
	Pharmacist	13
	Other professional	25
Works in primary care	Yes	56
	No	44
Age	30–39	31
	40–49	25
	50–59	25
	60+	19
Race	Alaska Native/American Indian	31
	White	63
	Other	13
Ethnicity	Not Hispanic	94
	Hispanic	0
	Unknown	6
Gender	Female	62
	Male	38
Customer-owners (n = 14)		%
Age	Range: 51–78 yrs, M = 63.2 (SD = 7.9)	
Data Collection Type	Focus group	62
	Individual interview	38
Race	Alaska Native/American Indian alone	71
	AN/AI and another race	29
Ethnicity	Hispanic	29
	Not Hispanic	71
Gender	Female	86
	Male	14
Highest level of education	Less than high school	21
	High school	29
	Any college	43
	Master’s degree	7

**Table 3:** Barriers and facilitators to ACP in general and implementing Jumpstart AN/AI.

SCF Employees (n = 16)	

Perceived Barriers	Perceived Facilitators

• Not enough time, i.e. short appointments with high demands • Staff shortages and turnover • Change fatigue • Hard to integrate electronic health record and patient portal • Documents sent home with customer- owners may not be returned • Employee discomfort talking about ACP • Customer-owner distrust in health care system • Low use of patient portal, especially among Elders • Customer-owners may not engage with ACP (e.g., overwhelmed, do not want)	• Primary care staff are familiar with process for ACP • Primary care providers are generally comfortable with having ACP conversations • High staff buy-in for ACP • Existing ACP fields in the EHR • Using technology to deliver services (e.g., EHR automation of Jumpstart) could create efficiency in health system • Staff can notarize ADs immediately • Integrated palliative care specialist available in primary care • Same-day appointments available • Community resource and behavioral health specialists can support ACP • Aligns with Existing Aging Well initiative • Customer-owners trust in health care system • Customer-owners are used to completing screeners and questionnaires

Customer-Owners (n = 14)	

Perceived Barriers	Perceived Facilitators

• Providers do not bring up ACP • Difficult to navigate between primary care and specialty care providers • Many customer-owners do not use patient portal • Some customer-owners may be reluctant to discuss ACP in groups • Customer-owners do not know about ACP • Lack of Alaska Native language interpreters in health system	• High trust in health care system • Strong relationships with primary care team • Some customer-owners use the patient portal • Customer-owners are comfortable with ACP conversations • Customer-owners are used to completing screeners and questionnaires • Support system for ACP • Support from AN/AI community

**Table 4:** Options for Jumpstart AN/AI implementation.

Option 1	Option 2

Questionnaire delivery: Offer multiple options Pencil and paper, tablet, patient portal, face to face with trusted team member Customer-owner-facing tip sheet: Provide in multiple formats	Questionnaire delivery: Customer-owner completes with pencil and paper with a trusted team member or is offered to take home with Your Care Your Choices materials and return Customer-owner-facing tip sheet: Upon questionnaire completion, immediately entered into EHR or scored, printed, and handed to Customer-owner and follow up appointment scheduled Provider-facing tip sheet: Available in the EHR or by email.
Printed immediately, mailed, patient portal document, secure email or message Provider-facing tip sheet: Available in the EHR or by email.	Ideally placed in ACP tab. Specific support person alerts provider to review prior to follow up appointment
Ideally placed in ACP tab Specific support person alerts provider to review prior to follow up appointment	

**Table 5:** Key decisions made for implementing Jumpstart AN/AI in the context of an RCT.

Implementation Needs	Key Decisions
Process for customer-owner to complete questionnaire	In-person, using either paper forms or on electronic tablets, facilitated by research team
Process for populating customer-owner tipsheet	Will be done in REDCap by research team
Process for populating provider tipsheet	Will be done in REDCap by research team
Process for sharing customer-owner tipsheet	Will be printed and shared immediately upon completion of survey
Process for sharing provider tipsheet	More information needed, will engage operational and clinical leaders in health system to determine at later date

## Data Availability

De-identified data and materials are available upon request from the project manager on this study at the Southcentral Foundation Ms. Shannon Medlock

## Abbreviations

ACP: Advance Care Planning
AD: Advanced Directive
AN/AI: America Native/American Indian
RCT: Randomized controlled trial
SCF: Southcentral Foundation
